# Adult axillary lymphangioma removal using indocyanine green fluorescence imaging system: A case report

**DOI:** 10.1016/j.ijscr.2020.05.090

**Published:** 2020-06-11

**Authors:** Osamu Kubota, Takashi Uchiyama, Koichi Nakamura, Yoshiro Hayashi, Yoshinori Onuki, Satoshi Baba

**Affiliations:** a Department of Surgery, Kikugawa General Hospital, Shizuoka, Japan; b Department of Diagnostic Pathology, Hamamatsu University School of Medicine, Hamamatsu, Japan

**Keywords:** ICG, indocyanine green, MMG, mammography, US, ultrasound, MRI, magnetic resonance imaging, PDE, photodynamic eye, NIR, near-infrared ray, Cystic lymphangioma, Indocyanine green fluorescence, Near-infrared fluorescence, Case report

## Abstract

**Introduction:**

Lymphangiomas are benign cystic tumors which arise from congenital malformations of the lymphatic system and are extremely rare in adulthood. We report a case of adult lymphangioma of the axilla that was removed after identifying the feeding lymphatic vessel using an indocyanine green (ICG) fluorescence imaging system.

**Presentation of case:**

A 35-year old woman presented to our hospital with a rapidly growing mass on her left axilla. She had been pregnant once before and delivered at 34 years of age. Mammography, ultrasonography, and magnetic resonance imaging revealed a tumor that consisted of multiple cysts, which led to a diagnosis of cystic lymphangioma. The ICG fluorescence imaging system indicated that only one lymphatic vessel, which was completely removed with ligation of the feeding lymphatic vessel, was flowing to the tumor. An immunohistological study demonstrated that the cystic endothelia were positive for podoplanin (D2-40), a marker of lymphatic vessels.

**Discussion:**

In addition to congenital factors, mechanical obstruction to lymphatic vessels by an external force, such as trauma or congestion of the lymphatic flow caused by increasing venous pressure during pregnancy or delivery might lead to lymphangioma in adulthood. Therefore, our patient’s pregnancy and delivery one year prior to discovery of the tumor seems to be the cause of her lymphangioma.

**Conclusion:**

Based on our findings, we recommend the complete excision to successfully treat adult-onset lymphangioma. We also suggest that visualization with ICG fluorescence imaging system is very useful for detecting the feeding lymphatic vessel and performing complete excision of the lymphangioma.

## Highlights

• Adult cystic lymphangioma is extremely rare.
• Diagnosis of lymphangioma is relatively easy based on the clinical image findings.
• Complete surgical excision is the most effective treatment for lymphangioma.
• The ICG fluorescence imaging system is very useful for performing complete excision.

## 1 Introduction

Lymphangiomas are benign malformations of the lymphatic system resulting from lymphatic dilatation with endothelial linings, which may be caused by congenital weakness of the wall, proliferation of the lymphatic vessels, and blockage of the lymphatic channels [1]. Lymphangiomas are usually found in the cervicofacial region (75 %) and axilla (20 %), and typically (in 90 % of cases) arise in infants who are less than 2 years old. Lymphangioma that develops in adulthood are therefore extremely rare [[2], [3], [4], [5], [6], [7], [8]].

The indocyanine green (ICG) fluorescence imaging system is a widely acceptable procedure for intraoperative detection of lymphatic vessels and sentinel lymph nodes [9].

We herein report a rare case of adult lymphangioma of the axillary region that was removed by means of the ICG fluorescence imaging system.

This work has been reported in line with the SCARE criteria [10].

## 2 Presentation of case

A 35-year old woman presented to our hospital with a rapidly growing egg-sized mass on the left axilla, detected by the patient one week prior. Of note, the patient had been pregnant once before and delivered at 34 years of age.

**Fig. 1:**
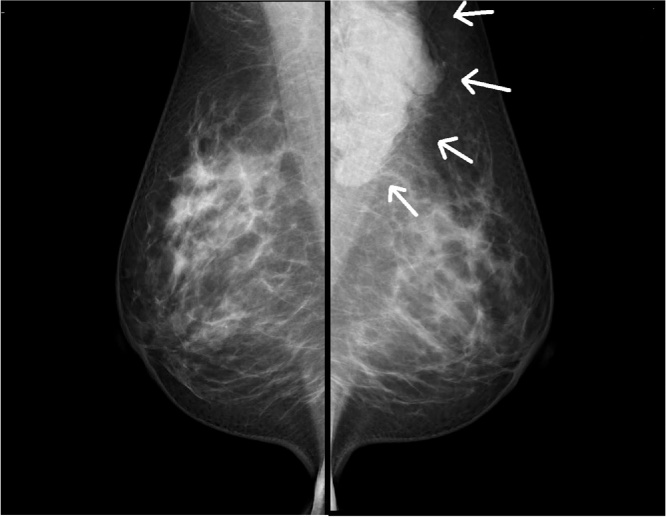
Mediolateral oblique mammogram. A lobulated circumscribed high-density mass of the left axilla (arrows).

**Fig. 2:**
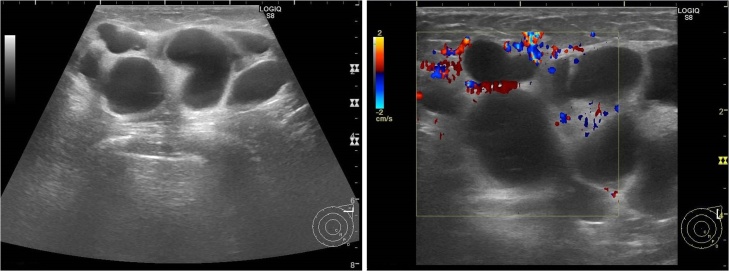
Ultrasound. Left: Lymphangioma consisting of multiple cysts and septa. Right: Color Doppler imaging indicating well flows in the walls and septa but not in the internal cysts.

**Fig. 3:**
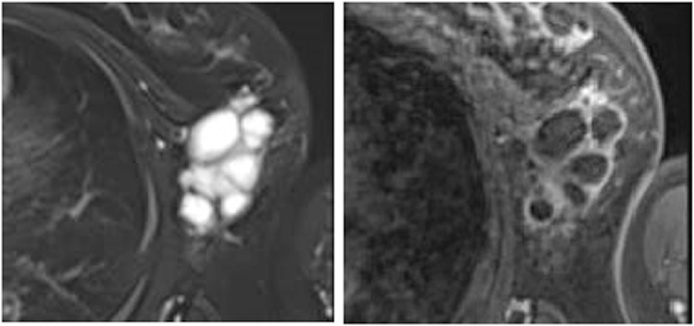
Magnetic resonance imaging (MRI). Left: High-intensity MRI of internal spaces on the T2-weighted image. Right: Enhancement of wells, surrounding walls, and internal septa on the T1-weighted dynamic image.

Physical examination revealed an elastic, soft, tender, and well-demarcated mass, 6.0 × 4.0 cm in size, and palpable in the left axilla. No skin changes were observed, including erythema, dimpling, nor retraction. A mediolateral oblique mammography (MMG) view indicated a lobulated circumscribed high-density mass, up to 8.0 cm in diameter, on the left axilla (Fig. 1). Furthermore, ultrasonography (US) revealed a multicystic anechoic lobulated tumor, 6.5 × 4.5 cm in size, in the left axilla, with connections between the multicystic structures. A posterior acoustic enhancement was detected. Obvious flows in the walls and septa were observed on color Doppler with no flows in the cystic spaces (Fig. 2). Magnetic resonance imaging (MRI) indicated that the internal spaces showed a high intensity on the T2-weighted imaging, while the surrounding walls and internal septa demonstrated a high intensity on the T1-weighted dynamic imaging (Fig. 3). Based on these findings, we diagnosed the patient with a cystic lymphangioma (hygroma) which required surgical excision.

**Fig. 4:**
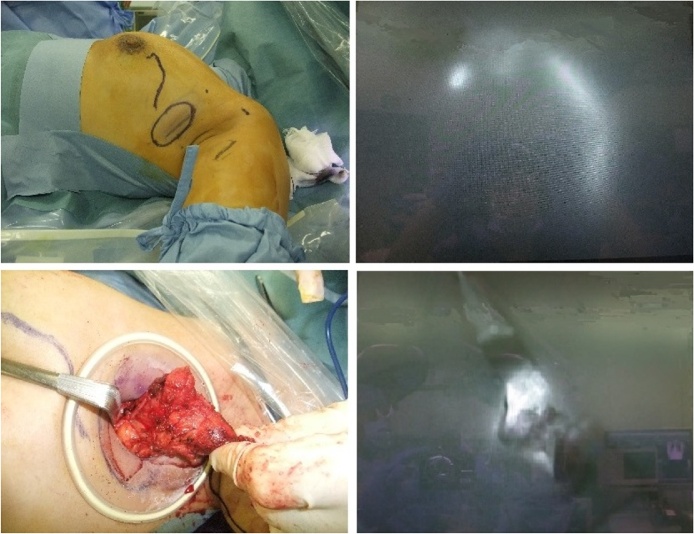
Intraoperative findings. Injection of ICG under the left areola. Left top: marking the lymphatic vessel and tumor with the naked eye. Right top: lymphatic vessel flowing into the tumor, visible with PDE. Left bottom: tumor removal with the naked eye. Right bottom: the tumor itself also glowing, visible with PDE.

A volume of 1.0 ml of indocyanine green (ICG, 0.5 mg/mL) was injected to the para-areolar subcutaneous area under general anesthesia, while the surgical field was observed with a near-infrared fluorescence imaging photodynamic eye (PDE) camera (Hamamatsu Photonics Co., Hamamatsu Japan). The fluorescent light indicated that only one lymphatic vessel was flowing into the tumor, while the tumor itself was glowing (Fig. 4). Subsequently, the tumor was removed with ligation of the feeding lymphatic vessel.

**Fig. 5:**
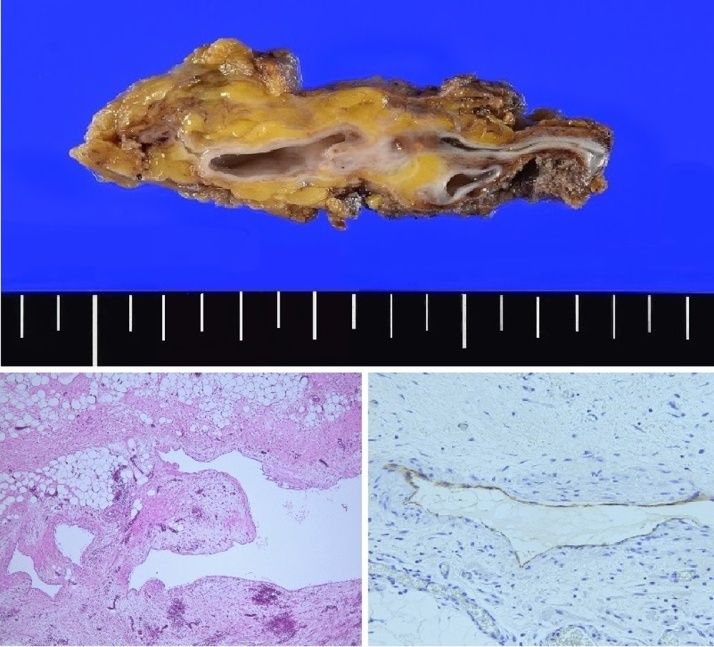
Macroscopic cut surface and histological findings. Top: a multicystic tumor, 6.5 × 2.5 cm in size, surrounding the fat tissue. Left bottom: cystic dilations lined by endothelia and surrounding fibrous and adipose tissue (40x original H&E magnification). Right bottom: endothelia positive for podoplanin (D2-40), a marker of lymphatic vessel (200x original magnification).

The removed tumor was 6.5 × 3.5 × 2.5 cm in size and consisted of multicystic structures with septa and surrounding walls. The histological and immunohistochemical studies revealed that the cystic walls and septa consisted of fibrous tissue associated with smooth muscle. Meanwhile, the endothelia were positive for podoplanin (D2-40), a marker of lymphatic vessels (Fig. 5). Therefore, histologically, the tumor confirmed macrocytic lymphangioma (lymphatic malformation). The postoperative course of the patient was uneventful and there were no signs of complications or recurrence.

## 3 Discussion

Lymphangiomas are classified into capillary (simple) and cystic (cavernous) based on their pathologic features. Capillary lymphangiomas are slightly elevated or sometimes pedunculated lesions between 1 and 2 cm in diameter, composed of networks of endothelium-lined spaces that are distinguishable from capillary channels. On the contrary, cystic lymphangiomas grow large up to 15 cm and are composed of massively dilated lymphatic spaces lined by endothelial cells and separated by intervening connective tissue stroma containing lymphoid aggregates [11].

In addition to congenital factors, such as remaining cystic dilatation of the lymphatic tissue or malformation of the lymphatic channels, mechanical obstruction to the lymphatic vessels by an external force, such as trauma or congestion of lymphatic flow caused by increasing venous pressure during pregnancy or delivery, might result in the appearance of lymphangiomas in adulthood [1]. Therefore, our patient’s pregnancy and delivery one year prior to presenting to our hospital seems to be the cause of her lymphangioma. The axilla contains fibro-adipose tissue, the axillar artery and vein, a brachial plexus, axillary lymph nodes and associated lymphatics, and an accessory breast. Therefore, previous studies list hemangioma, schwannoma, lipoma, accessory breast tumor, and benign or malignant lymphadenopathy as differential diagnoses of lymphangioma [12]. However, due to findings including a large circumscribed lobulated high-density mass, well-defined anechoic cysts with septa, hyperintense cystic spaces, and enhanced walls and septa observed using MMG, an US, and T1- and T2-weighted imaging, respectively, allowed for an easy diagnosis of lymphangioma [13,14].

Whereas spontaneous resolution of lymphangioma is uncommon [15], sclerotherapy, which includes injection of sclerosing agent such as bleomycin or streptococcal lysin (OK-432), is an acceptable treatment option in infants, but may lead to complications such as discoloration of the injection site, sudden growth of the lymphangioma, fever, vomiting, cellulitis, interstitial pneumonia, and pulmonary fibrosis [16]. Therefore, complete surgical excision of the tumor is the most favorable treatment of lymphangioma, especially in adults.

The ICG fluorescence imaging system is a new technique for intraoperative navigation, widely utilized for intraoperative visualization of lymphatic vessels, sentinel lymph nodes, tumors, bile ducts, and tissue perfusion [17]. Near-infrared ray (NIR) light (700−900 nm) is more advantageous than visible light due to its capability to penetrate deeper into tissues, up to 10 mm for in vivo visualization. ICG as a NIR fluorescent agent (fluorophore) generate excitation light with a wavelength between 750 nm and 810 nm when NIRs are exposed. As NIR fluorescence cannot be directly visualized with the naked eye, it requires confirmation on the monitor in real-time by means of NIR fluorescence imaging system PDE [18,19].

## 4 Conclusion

Based on the findings from this study, we recommend the complete excision of the tumor to successfully treat adult-onset lymphangioma. Moreover, we suggest that ICG fluorescence imaging system is very useful for intraoperative navigation and visualization of the lymphatic vessel and tumor, which in turn aid in the complete excision of the lymphangioma.

## Declaration of Competing Interest

There are no conflicts of interest.

## Sources of funding

This research did not receive any specific grant from funding agencies in the public, commercial, or not-for-profit sectors.

## Ethical approval

This case report is exempt from ethical approval in our institution.

## Consent

Written informed consent was obtained from the patient for publication of this case report and accompanying images. A copy of the written consent is available for review by the Editor-in-Chief of this journal on request.

## Author contribution

Osamu Kubota: medical practitioner, a surgeon who operated on patient and contributed to writing the paper.

Takashi Uchiyama and Koichi Nakamura: surgeons who operated on patient and contributed to writing the paper.

Yoshiro Hayashi and Yoshinori Onuki: provide assistance for medical practices and contributed to writing the paper.

Satoshi Baba: a pathologist who contributed on the pathological section of the paper.

## Registration of research studies

Registration is not applicable, because this manuscript is a case report, not a research study.

## Guarantor

Osamu Kubota

## Provenance and peer review

Not commissioned, externally peer-reviewed.
